# Cohort Profile: The Green and Blue Spaces (GBS) and mental health in Wales e-cohort

**DOI:** 10.1093/ije/dyac080

**Published:** 2022-04-21

**Authors:** Daniel A Thompson, Rebecca S Geary, Francis M Rowney, Richard Fry, Alan Watkins, Benedict W Wheeler, Amy Mizen, Ashley Akbari, Ronan A Lyons, Gareth Stratton, James White, Sarah E Rodgers

**Affiliations:** Population Data Science, Swansea University Medical School, Faculty of Medicine, Health and Life Science, Swansea University, Swansea UK; Department of Public Health, Policy and Systems, University of Liverpool, Liverpool, UK; European Centre for Environment and Human Health, University of Exeter Medical School, Knowledge Spa, Royal Cornwall Hospital, Cornwall, UK; Population Data Science, Swansea University Medical School, Faculty of Medicine, Health and Life Science, Swansea University, Swansea UK; Population Data Science, Swansea University Medical School, Faculty of Medicine, Health and Life Science, Swansea University, Swansea UK; European Centre for Environment and Human Health, University of Exeter Medical School, Knowledge Spa, Royal Cornwall Hospital, Cornwall, UK; Population Data Science, Swansea University Medical School, Faculty of Medicine, Health and Life Science, Swansea University, Swansea UK; Population Data Science, Swansea University Medical School, Faculty of Medicine, Health and Life Science, Swansea University, Swansea UK; Population Data Science, Swansea University Medical School, Faculty of Medicine, Health and Life Science, Swansea University, Swansea UK; Department of Sport and Exercise Sciences, Applied Sports Technology, Exercise and Medicine A-STEM Research Centre, School of Engineering and Applied Sciences, Faculty of Science and Engineering, Swansea University, Swansea UK; Centre for Trials Research, School of Medicine, Cardiff University, Cardiff, UK; Department of Public Health, Policy and Systems, University of Liverpool, Liverpool, UK

Key features

- The Green Blue Spaces (GBS) e-cohort includes 2.8 million UK adults (2008-19) and was established to quantify the impact of natural environments on mental health and wellbeing in Wales, UK.
- This is the first e-cohort with national household-level longitudinal environment metrics (annual) for 1.4 million residences linked to longitudinal electronic health records (updated quarterly), with a subgroup of 5312 linked survey responses on visits to outdoor spaces and wellbeing.
- Baseline and follow-up information was extracted quarterly through electronic record linkage, including mental health service use and sociodemographic and economic characteristics.
- After almost 12 years’ follow-up, 0.7% were lost to follow-up due to migration out of Wales and were replaced with in-migration and those reaching the age of 16 years (25%), 9.9% died and 28% had at least one common mental health episode recorded with their general practitioner (GP).
- The GBS e-cohort uses a controlled data-access model [https://saildatabank.com/application-process/].

## Why was the cohort set up?

The Green Blue Spaces (GBS) e-cohort, funded by the National Institute for Health Research (NIHR), was established to understand the impact of green and blue spaces (GBS) on mental health and wellbeing.^1^ The importance of GBS for mental health has been highlighted particularly during the COVID-19 pandemic.^2^ We processed open-source environmental data and Ordnance Survey data to create residence-level, longitudinal environment metrics for Wales, UK. These were linked to anonymised, administrative, routinely collected National Health Service (NHS) electronic health records. The cohort has individual-level linkage to a subgroup who were surveyed (cross-sectionally) to examine the association between visits to GBS and wellbeing. The size of the cohort allows examination of associations within and between subgroups not limited to socioeconomic disadvantage.

Living close to GBS such as parks, woodlands, trails, ponds, lakes, rivers and beaches is associated with positive impacts on physical and mental health.^3–6^ However, the majority of evidence (cross-sectional) has not unpicked associations between the type, proximity, quantity and ‘qualities’ of GBS, and changes in mental health/wellbeing.^7^^,^^8^ As a result, existing evidence to inform policies shaping our environment is limited.^9–11^ In the first 3 years, the cohort will provide policy-relevant results on these associations^1^ to inform evidence-based public health, planning and regeneration decisions on the protection, development and management of GBS to promote and protect health and wellbeing.

## Who is in the cohort?

The GBS cohort is held in the Secure Anonymised Information Linkage (SAIL) Databank,^12^ a trusted research environment providing secure, privacy-protecting storage of anonymised, person-based, demographic, health, social and education data for the population of Wales.^13^^,^^14^ The cohort is constructed using data from the Welsh Demographic Service Dataset (WDSD). This dataset contains demographic characteristics of everyone registered with a general practitioner (GP) in Wales, providing data to the SAIL databank (80% population coverage^15^). It is used as the primary population register in the SAIL Databank. The WDSD contains the names and addresses with from-to dates of residency in each home; these are updated when patients inform their GP they have moved home. Researchers accessed an anonymised version of the WDSD, and calculated residency dates in each home and also house moves. All members of the household are included in the cohort, with individuals nested within each household.

The demographic dataset was used as the population spine, with additional data linked as follows:

- Welsh Longitudinal General Practice (WLGP): information on symptoms, diagnoses, prescriptions, and referrals^1^;
- Annual District Death Extract from the Office of National Statistics (ONS) mortality register^2^;
- Welsh Index of Multiple Deprivation (WIMD), the Welsh Government’s official measure of relative deprivation for small areas in Wales^3^;
- Rural-urban ONS classifications at Lower Layer Super Output Area (LSOA)^4^;
- National Survey for Wales (NSW), an annual, repeated, cross-sectional survey of about 12 000 adults in Wales (2016-17^16^ and 2018-19^17^ surveys) including responses on wellbeing and visits to outdoor spaces.

**Figure 1:**
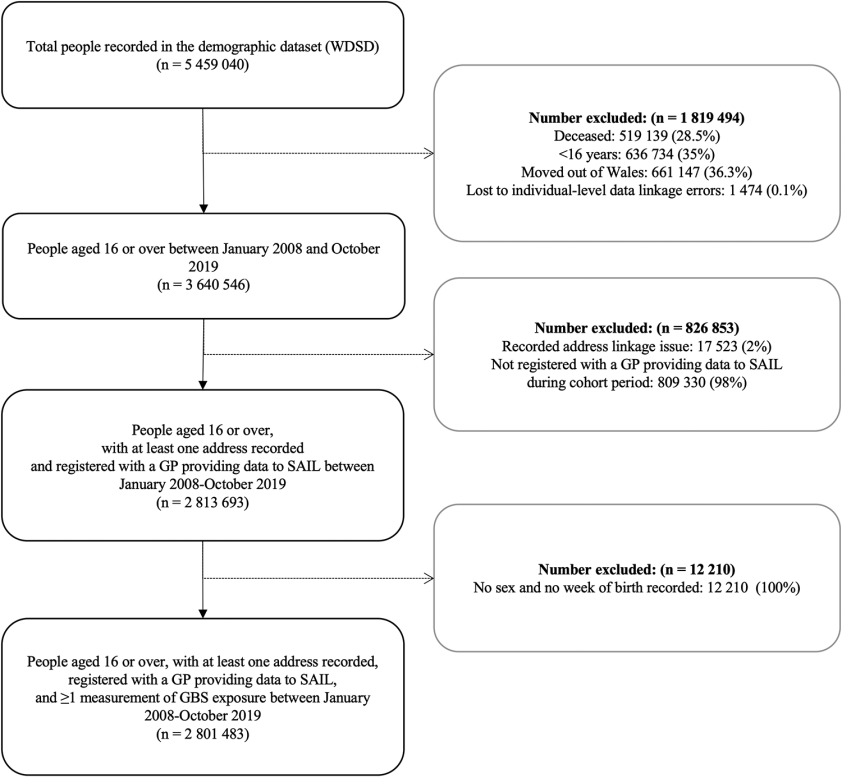
Cohort enrolment using the demographic dataset (WDSD) following linkage to the Welsh Longitudinal General Practice (WLGP) dataset. SAIL, Secure Anonymised Information Linkage; GP, general practice; GBS, Green Blue Spaces.

The cohort comprises 2 801 483 individuals—all persons aged 16 and over registered with a practice providing GP records to the SAIL Databank. We intentionally removed people who did not fit with the cohort criteria (Figure 1). We excluded 839 063 individuals who had missing data, e.g. they were not registered with a GP providing data to the SAIL Databank, did not have a Welsh residential address between January 2008 and October 2019 or did not have sex or week of birth recorded in WDSD.

We created measures of GBS exposure and access for all homes in Wales, using several environmental datasets: (i) satellite data (Landsat TM^18–21^ 2008–19) to create annual greenness densities of the mean Enhanced Vegetation Index (EVI) and Normalised Difference Vegetation Index (NDVI) within 300 m of each residence; (ii) Ordnance Survey MasterMap Topography Layer^22^ (2018) to capture natural and man-made features, including the outline of homes and parks; (iii) Ordnance Survey MasterMap-derived Greenspace dataset (2018)^23^; (iv) local authority (LA) technical advice notes, legally required records of data on sport, recreation and open spaces managed by local authorities (LAs); (v) open source portal data from Lle (forestry, urban tree cover)^22^; and (vi) OpenStreetMap road/footpath data.^24^ Environmental data were linked to the cohort at individual-level data, using a residential version of the split file linkage process.^25^^,^^26^ A final GBS typology (Supplementary Table S1, available as Supplementary data at *IJE* online) was used to create GBS access metrics for each home in Wales.

A cohort subgroup responded to Natural Resources Wales (NRW) questions in the 2016–17 and 2018–19 National Survey for Wales (NSW).^16^^,^^17^ The NSW is an annual repeat, cross-sectional, government-sponsored, omnibus survey of a representative sample of the population of Wales (annual *n* ∼12 000). Topics include education, culture, health and wellbeing and more detailed information on socioeconomic circumstances than administrative data. The NRW questions (sub-sample, *n *= 5312)^27^^,^^28^ record whether respondents visited outdoor spaces in Wales, including time spent outdoors on leisure activities, and types of activities undertaken. NSW respondents aged ≥16 years, who consented to NSW-administrative data linkage (>90%), were linked to the cohort.

We derived environmental metrics for all potential residences in Wales (*n* = 1 498 120). Of these, 1 179 817 (78%) residences were linked to the cohort through the WDSD. There were 318 303 unlinked potential homes (likely holiday homes, caravans, guest-houses), either because they did not match an address of an individual registered with a GP in Wales or were inhabited by people not registered at a GP practice. Area-level characteristics of residences linked and unlinked to the cohort were compared to check for potential bias (see ‘What has it found?’). Of the 2 801 483 individuals in the cohort, 622 025 (22.2%) moved home once between 2008 and 2019, and 567 877 (20.3%) moved home more than once. Exposures and outcomes are extracted/updated quarterly.

## How often have they been followed up?

Health-related outcomes were extracted quarterly. Environmental metrics were calculated annually but updated quarterly if cohort members moved home (see ‘What has been measured’). The dynamic cohort design allows new people to enter the cohort each quarter as they reached age 16 years or moved into Wales. Cohort sample size in each quarter is provided in Supplementary Table S2 (available as Supplementary data at *IJE* online). The current linkage of environmental and administrative data sources ended in September 2019, creating an 11-year cohort with annual follow-up for all, and quarterly follow-up for people moving home. Non-environmental datasets are routinely updated in SAIL, enabling health outcomes for the cohort to be followed up for longer. A total of 5 791 cohort members completed NRW questions in the 2016-17 and 2018-19 NSW. Further waves of the NSW have been consented for data linkage in SAIL.

The GBS e-cohort cohort was created from multiple data sources with varying levels of completeness across different variables. Known exclusions, due to missing data on age or sex (0.4%) or at least one primary environmental measure (EVI, <0.01%), resulted in a cohort of 2 801 483 people (Figure 1). This cohort has 24.9 million-person-years of follow-up. An additional average of 30 238 people joined the cohort annually through migration into Wales or reaching age 16 years (∼34 709 people annually), totalling 710 570 (25%). Annually, an average of 22 987 people died and 1 603 permanently moved out of Wales, totalling 294 437 (10.5%).

## What has been measured?

Cohort variables are presented in themes: (i) sociodemographic and economic characteristics; (ii) common mental health disorders/wellbeing; (iii) comorbidity index; (iv) social environment and life events (births/deaths in the household); (v) environmental metrics; and (vi) other administrative cohort information (Table 1).

**Table 1:** List of cohort variables available

Domain	Sub-domain	Individual (I)/Residence (R) level
i. Sociodemographic and economic characteristics	Age	I
	Sex	I
	Deprivation^a^	R
	Rurality	R
ii. Common mental health disorders/wellbeing	Depression	I
	Anxiety	I
	Common Mental Disorder (CMD)	I
	Warwick-Edinburgh Mental Wellbeing Scale (WEMWBS)^c^	I
	Office for National Statistics (ONS4) measures of wellbeing`	I
iii. Comorbidity index/hospital episode count	Modified Charlson Co-morbidity Index^b^	I
	Inpatient hospital episode^d^	I
iv. Social environment and life events	Birth in household	R
	Death in household	R
	Household composition (count of children <16 in household)	R
	Time since last residential move	I
v. Environmental metrics	Enhanced Vegetation Index (EVI)	R
	Normalized Difference Vegetation Index (NDVI)	R
	Access to GBS (distance/size/type)	R
	GBS visiting behaviour (from National Survey for Wales)	I
vi. Other administrative cohort information	Cohort entry/exit reason (death/migration)/date	I
	Anonymised Linkage Field (ALF)^e^	I
	Residential Anonymised Linkage Field (RALF) with from/to dates^e^	R
	Lower layer Super Output Area (LSOA)	R

a 2011 and 2014 Welsh Index of Multiple Deprivation (WIMD) as defined by the Welsh Index of Multiple Deprivation (IMD) quintiles 2011 and 2014,^29^.

b Charlson Comorbidity Index as defined by Charlson *et al.*^30^

c NSW respondents only.

d inpatient hospital episode as identified in Patient Episode Database for Wales (PEDW);

e Anonymised Linking Field (ALF) and Residential Anonymised Linking Field (RALF) are individual and household anonymised linking fields, respectively, within the Secure Anonymised Information Linkage (SAIL) Databank.^31^^,^^32^

Key health metrics are (quarterly): Common Mental Health Disorder (anxiety and depressive disorders) and a count of all GP events (extracted from WLGP). The WLGP is collated from clinical information systems in use at each general practice around Wales, and uses Read codes recorded during a GP consultation. Test results are electronically transferred into the WLGP from secondary care systems. To identify people with Common Mental Health Disorders (CMDs), we applied an existing validated prevalence algorithm with high sensitivity to detect cases of CMD (anxiety and depression).^33^ We identified people with CMD each quarter when they had either a historical diagnosis(es) currently treated, and/or current diagnoses or symptoms (treated or untreated) from Read codes (detailed in Supplementary Table S3, available as Supplementary data at *IJE* online) in their GP record in the WLGP data (Algorithm 10).^33^ The algorithm identifies ‘current’ diagnoses/symptoms as relevant Read codes in the preceding 1-year period. It identifies ‘historical’ diagnoses through a search for relevant Read codes through the cohort data outside the ‘current’ period. The length of retrospective data available varied between individuals in the cohort, depending on the length of their registration with a GP supplying data to SAIL. CMD treatment was identified as at least one prescription for an antidepressant, anxiolytic or hypnotic in the 1-year current period.^1^ We did not include cognitive behavioural therapies or other non-drug treatments in our CMD case definition, as this information was not available in WLGP. The algorithm applied to identify probable cases of CMD has high specificity and positive predictive value for detecting CMD (anxiety and depression) but, as expected, has low sensitivity.^33^ We identified adults (16+ years) with CMD in the GP dataset. We refer to people ‘having a CMD’, but we acknowledge that this only captures those who have sought care for their CMD in primary care. Community prevalence will be significantly higher, because only about one-third of people affected by CMD seek help in primary care.^4^ GP-specific events were converted from daily counts to a binary variable and then aggregated to quarterly counts. This eliminated counting multiple test results. Each individual in the cohort also had quarterly measures for Charlson comorbidity index^30^ and a count of hospital admissions.

### Environmental metrics

GBS exposure within 300 m of each home in Wales was measured yearly from open source satellite imagery. Three variables representing ambient green/blueness were linked to the cohort:

- mean EVI (minimum, mean, median, max);
- mean Normalized Difference Vegetation Index (NDVI) (minimum, mean, median, max);
- coastal and/or inland water (yes/no);

We used imagery with less than 20% cloud cover to estimate EVI/NDVI, resulting in 87.7% of homes with full coverage of EVI and NDVI values from 2008 to 2019. Where homes were missing an EVI/NDVI value for a given year, and neighbouring years were available, we imputed these values.

The potential for an individual to access a range of types (Supplementary Table S1) of GBS, along a network of paths and roads within 1600 m of each home, was modelled for 2012 and 2018. Ambient green/blueness, and potential to access GBS, were augmented by survey responses about leisure time visits to outdoor spaces in Wales for the NSW subgroup.

Household-individual data linkage methods created a longitudinal dataset with the potential for a granular temporal examination of the impact of changes in green and blue space on health inequity for individuals. This design is more appropriate than previous studies for inferring causal links.^1–3^ Cohort members have their home location linked to appropriately synchroniezd environmental data, extracting subsequent health outcomes from their electronic health records. This provides the opportunity to construct natural experiments or pragmatic trials within the cohort^5^^,^^6^.

## What has it found?

Using a combination of open source environmental and national mapping agency data, we have demonstrated the feasibility of creating individual-level, longitudinal, environment exposure data with national coverage for 2.8 million adults in Wales (2008–19). Longitudinal linkage of national-level environmental data, for 1.4 million homes with routinely collected electronic health records and socioeconomic data, allows this cohort to be used to assess the impact of a changing environment on subsequent common mental health disorders, wellbeing and other health outcomes.^26^

At an individual level, there was little variation in data completeness between those identified as having a CMD at least once and those without having a CMD: 99.9% (*n* = 816 020) and 99.4% (*n *= 1 983 590), respectively. At a household level, 92.3% (*n* = 2 598 211) of the cohort were linked to a home address for every quarter they were in the e-cohort. Individuals were censored during a quarter if no place of residence could be linked, or if their GP did not provide data to the databank. Individuals with at least one CMD episode had 90.4% (*n* = 739 054) residential data completeness compared with 93.1% (*n* = 1 859 157) of those without a CMD.

Full environmental data (EVI and NDVI) were linked for 85% of the cohort (*n* = 2 384 489) for their complete cohort duration. We examined the linkages to check for bias by deprivation and rurality. The percentage of unlinked homes did not increase with deprivation. However, we found that a higher proportion of unlinked homes were in rural areas. We did not find a systematic bias with EVI; mean EVI for unlinked and linked homes were similar (0.3, Table 2).

**Table 2:** Area-level deprivation and settlement type, overall and by mean ambient exposure (mean EVI) of residences linked and unlinked to the e-cohort

Group		All		Linked to cohort		Not linked	
		*n*	Column %	*n*	Column %	*n*	Column %
Welsh Index of Multiple Deprivation (WIMD) quintiles	Most deprived	292 733	19.5	*243 928*	20.7	48 805	15.3
	Next most deprived	302 100	20.2	248 265	21.0	53 835	16.9
	Mid-deprived	315 169	21.0	241 919	20.5	73 250	23.0
	Next least deprived	309 795	20.7	219 215	18.6	90 580	28.5
	Least deprived	278 323	18.6	226 490	19.2	51 833	16.3
ONS settlement type^40^	Rural town and fringe	197 499	13.2	161 417	13.7	36 082	11.3
	Rural town and fringe in a sparse setting	69 875	4.7	42 346	3.6	27 529	8.6
	Rural village and dispersed	101 978	6.8	70 118	5.9	31 860	10.0
	Rural village and dispersed in a sparse setting	127 178	8.5	80 361	6.8	46 817	14.7
	Urban city and town	973 872	65.0	802 972	68.1	170 900	53.7
	Urban city and town in a sparse setting	27 718	1.9	22 603	1.9	5115	1.6

Mean EVI		All		Linked to cohort		Unlinked	
		Mean	SD	Mean	SD	Mean	SD
All		0.30	0.13	0.30	0.12	0.30	0.12
Welsh Index of Multiple Deprivation (WIMD) quintiles	Most deprived	0.25	0.10	0.26	0.10	0.22	0.11
	Next most deprived	0.28	0.11	0.28	0.11	0.27	0.13
	Mid-deprived	0.32	0.14	0.31	0.14	0.33	0.16
	Next least deprived	0.33	0.15	0.32	0.14	0.36	0.16
	Least deprived	0.31	0.11	0.31	0.11	0.33	0.13
ONS settlement type^40^	Rural town and fringe	0.32	0.11	0.32	0.11	0.33	0.12
	Rural town and fringe in a sparse setting	0.33	0.13	0.33	0.14	0.33	0.13
	Rural village and dispersed	0.42	0.14	0.42	0.14	0.43	0.14
	Rural village and dispersed in a sparse setting	0.45	0.15	0.44	0.16	0.45	0.15
	Urban city and town	0.26	0.10	0.27	0.10	0.25	0.11
	Urban city and town in a sparse setting	0.27	0.13	0.28	0.13	0.24	0.14

ONS, Office of National Statistics; EVI, Enhanced Vegetation Index.

A total of 29% of the cohort (816 242) sought care for a CMD in general practice between January 2008 and October 2019. A total of 461 728 (16%) people in the cohort had a previously diagnosed CMD for which they sought care in general practice, subsequently entering the e-cohort (‘historical diagnosis’). For the more than 300 000 people newly seeking treatment for a CMD from their GP (i.e. who had no ‘historical diagnosis’, *n* = 305 779), a larger proportion (14%, *n* = 43 350) were living in more affluent, greener areas (measured by mean EVI) by the end of their time in the cohort (relative to when they entered the cohort) compared with only 8% (*n* = 23 795) who were living in deprived areas with less greenery immediately surrounding the home. In contrast, most people (75%, *n* = 267 446) who had a ‘historical’ CMD diagnosis and who also had a CMD during the cohort period (2008-19, *n *= 358 126), lived in greener areas by the end of their time in the cohort.

People living in the most deprived areas had on average less ambient greenness around their home than those living in the least deprived areas (mean EVI 0.25 vs 0.31, respectively, Table 2). The dynamic cohort captures abrupt GBS changes resulting from home moves as well as *in situ* slower changes in ambient greenness. More than one-fifth (22.6%) of the adult population in the most deprived quintile moved home at least once during the cohort period, with fewer moving in the least deprived (18.7%) and next-least deprived (18.2%) quintiles (Table 3). Younger people (<30 years old) and those living in the most deprived areas had the highest prevalence of moving at least once during their time in the cohort (48.9% and 22.6%, respectively, Table 3).

**Table 3:** Sociodemographic characteristics of the cohort at baseline with mean EVI by age, deprivation and sex

Group		Cohort		Moved home at least once		Ambient exposure	
		(*n*)	(%)	(*n*)	(%)	Mean	SD
Sex	Male	1 381 576	49.3	561 868	47.2	0.29	0.09
	Female	1 419 907	50.7	628 034	52.8	0.29	0.09
Age group	16–21	614 265	21.8	316 803	26.6	0.29	0.1
	22–30	418 046	14.9	264 988	22.3	0.27	0.09
	31–40	405 553	14.1	201 099	16.9	0.29	0.09
	41–50	409 772	14.6	149 919	12.6	0.3	0.09
	51–60	353 182	12.6	101 296	8.5	0.31	0.09
	61–70	303 247	10.8	68 420	5.8	0.31	0.09
	71–80	190 964	6.8	47 581	4	0.29	0.09
	81+	106 482	3.8	39 796	3.3	0.32	0.14
Welsh Index of Multiple Deprivation (WIMD) quintiles	Most deprived	568 394	20.8	254 944	22.6	0.26	0.08
	Next most deprived	544 315	19.9	229 384	20.4	0.28	0.08
	Mid-deprived	559 434	20.5	226 951	20.1	0.31	0.1
	Next least deprived	508 838	18.6	205 130	18.2	0.32	0.11
	Least deprived	552 939	20.2	210 323	18.7	0.3	0.08
ONS settlement type	Urban	1 847 233	68.2	778 507	69.9	0.21	0.08
	Town and fringe	452 951	16.7	181 507	16.3	0.26	0.1
	Rural	408 559	15.1	154 125	13.8	0.35	0.13

Baseline is defined as the first period an individual enters the cohort.

ONS, Office of National Statistics.

We will apply advanced analytical approaches to the longitudinal health and exposure cohort, with the aim of quantifying the impact of GBS on individual-level mental health and wellbeing.^1^ The use of routinely collected historical data and established linkage mechanisms allows this e-cohort to be extended, either to include those under 16 years and/or to evaluate the impact of natural environments on further health, social and public health outcomes. Published cohort papers are listed at [https://fundingawards.nihr.ac.uk/award/16/07/07]. As part of the National Institute for Health Research (NIHR) School for Public Health Research, a doctoral fellowship has been awarded to use the cohort (September 2022-September 2027), with proposal title: Longitudinal analysis of the impact of green and blue spaces on health.

## What are the main strengths and weaknesses?

The cohort is subject to minimal attrition due to the inclusion of all GP-registered individuals, unless individuals have opted out by making a request to their GP (see https://saildatabank.com/faq/). This minimizes the potential for selection bias. The cohort currently contains 2 801 483 adults. This will change with further follow-up years because the dynamic e-cohort structure accommodates migration in and out of Wales, as well as deaths and ageing into the cohort (i.e. reaching age 16 years). This large adult population cohort provides sufficient power to examine variations between subgroups to investigate inequalities.

We reduced ecological fallacy using privacy-protecting data linkage methods to construct household measures of GBS.^5^^,^^6^ Longitudinal environmental metrics, and linkage methods, enable an objective assessment of environmental changes, with no research burden for individuals.^34–36^

A strength of this cohort is the ability to disentangle health outcomes from ‘greening gentrification’ by anonymously ‘tracking’ individuals over time.^37^ System-wide natural changes may be slowly evolving and so the impact on population health requires longer follow-up. Over a long duration, place-based improvements may displace an area’s original population with those who are more affluent and healthier (‘gentrification’). Results of place-based intervention studies investigating area-level health effects over long periods of time are therefore likely to record health outcomes of a different, healthier, population.

Like other electronic health records cohorts, the GBS e-cohort data are predominantly routinely recorded and lack data on behaviour, some potential confounding factors and outcomes such as wellbeing. There is no health-related quality of life instrument routinely used to assess changes in health status in general practice in Wales. The cohort is largely restricted to detecting changes in outcomes that involve health service use. However, through linkage to survey data, a subset of the cohort has information on wellbeing as well as on behaviours such as time spent visiting GBS (*n* = 5312 adults).

The validity and reliability of research using routinely collected data depend upon its quality and completeness. Overall, the validity of primary care diagnoses in the UK tends to be high.^38^ Case-finding for CMD in routinely collected administrative health data can unobtrusively identify patients for mental health research, including on the effects of intervention.^39^ Diagnostic coding can differ between clinicians/practices over time, which may influence the sensitivity and specificity of algorithms to identify patients using a specific case definition in e-cohorts over time. A validation study, comparing using Read codes and algorithms for CMD case-finding (including the algorithm we have used) with the five-item Mental Health Inventory, demonstrated that using diagnosis and current treatment alone to identify CMD using routinely collected GP data would miss a number of true cases, given changes in GP recording behaviour between 2000 and 2010. Including historical diagnoses with current treatment and symptoms, as in this cohort, increases sensitivity.

We captured annual ambient exposure to greenness, and temporally matched these to subsequent health outcomes. This improves on previous studies that did not have the data or systems to achieve this. We were unable, however, to continue this with the access metrics because several key data sources were not updated frequently and do not currently capture change in land use consistently. This has created a temporal mismatch between (annual) greenness measures (EVI, NDVI) and access measures (2018), which means we could not allocate a precise period when access to a GBS (new or old) may have changed. We recommend that GBS data providers update data regularly using consistent standards to capture changes in access to, and quality of, GBS through time.

## Can I get hold of the data? Where can I find out more?

This cohort is stored and maintained in the SAIL Databank at Swansea University, Swansea, UK. This is a controlled access cohort; all proposals to use SAIL data are subject to review by an independent Information Governance Review Panel. Where access is granted, it is gained through a privacy protecting safe haven and remote access system (SAIL Gateway). The cohort data will be available to external researchers for collaborative research projects after 2022. For further details about accessing the cohort, contact [saildatabank.com] and Sarah Rodgers [ARCNWC@liverpool.ac.uk] for opportunities to collaborate with the original investigator team.

## Ethics approval

This cohort is based on routinely collected administrative, environment and survey data. All data will be anonymised into a secure databank, and therefore there will be no mechanism for informing potential cohort participants of possible benefits and known risks. The cohort received approval from an independent Information Governance Review Panel, an independent body consisting of membership from a range of government, regulatory and professional agencies. We obtained informed consent to use the linked and anonymised NSW data within the SAIL databank. All routinely collected anonymised data held in SAIL are exempt from consent due to the anonymised nature of the databank (under section 251, National Research Ethics Committee).

## Data availability

See ‘Can I get hold of the data?’, above.

## Supplementary data

Supplementary data are available at *IJE* online.

## Author contributions

S.E.R. designed and led the development of the cohort. D.T. produced the analysis and cohort linkage and drafted the paper with R.G. R.F. and A.M. produced the exposure metrics and reviewed the paper. A.W. provided input on analytical strategy. F.R. and B.W. produced the analysis and linkage for individuals linked to NSW survey and reviewed the paper. R.L., G.S. and A.A. reviewed the paper. All authors contributed to cohort design through input to regular meetings. All authors reviewed the final submitted paper.

## Funding

The GBS and Mental Health in Wales cohort was developed as part of independent research funded by the National Institute for Health Research (NIHR), project number 16/07/07, and the UK Prevention Research Partnership, GroundsWell (MR/V049704/1). The views expressed are those of the author(s) and not necessarily those of the NHS, the NIHR or the Department of Health and Social Care.

## Supplementary Material

dyac080_Supplementary_Data
