# Supplementary material for: Cohort Profile: The Green and Blue Spaces (GBS) and mental health in Wales e-cohort
Source: Int J Epidemiol. 2022 Apr 21;51(5):e285–94. doi: 10.1093/ije/dyac080 (PMC9558062; doi:10.1093/ije/dyac080)
Supplement: dyac080_Supplementary_Data [file dyac080_supplementary_data.zip › ije-2021-07-1108-File005.docx]

Supplementary Table S2: Cohort size through time

| **Year** | **Quarter Start Date** | **n** |
| --- | --- | --- |
| 2008 | 01/01/2008 | 2103111 |
|  | 01/04/2008 | 2107408 |
|  | 01/07/2008 | 2111701 |
|  | 01/10/2008 | 2117704 |
| 2009 | 01/01/2009 | 2118899 |
|  | 01/04/2009 | 2121422 |
|  | 01/07/2009 | 2125537 |
|  | 01/10/2009 | 2125393 |
| 2010 | 01/01/2010 | 2125026 |
|  | 01/04/2010 | 2127224 |
|  | 01/07/2010 | 2129044 |
|  | 01/10/2010 | 2128770 |
| 2011 | 01/01/2011 | 2124844 |
|  | 01/04/2011 | 2127994 |
|  | 01/07/2011 | 2129393 |
|  | 01/10/2011 | 2132760 |
| 2012 | 01/01/2012 | 2133680 |
|  | 01/04/2012 | 2133763 |
|  | 01/07/2012 | 2138596 |
|  | 01/10/2012 | 2139588 |
| 2013 | 01/01/2013 | 2124985 |
|  | 01/04/2013 | 2137159 |
|  | 01/07/2013 | 2140857 |
|  | 01/10/2013 | 2141313 |
| 2014 | 01/01/2014 | 2135564 |
|  | 01/04/2014 | 2140349 |
|  | 01/07/2014 | 2136514 |
|  | 01/10/2014 | 2138878 |
| 2015 | 01/01/2015 | 2136163 |
|  | 01/04/2015 | 2136043 |
|  | 01/07/2015 | 2135615 |
|  | 01/10/2015 | 2130217 |
| 2016 | 01/01/2016 | 2128623 |
|  | 01/04/2016 | 2131122 |
|  | 01/07/2016 | 2133666 |
|  | 01/10/2016 | 2132855 |
| 2017 | 01/01/2017 | 2130525 |
|  | 01/04/2017 | 2119523 |
|  | 01/07/2017 | 2120825 |
|  | 01/10/2017 | 2118685 |
| 2018 | 01/01/2018 | 2107282 |
|  | 01/04/2018 | 2100101 |
|  | 01/07/2018 | 2085570 |
| 2019 | 01/10/2018 | 2078215 |
|  | 01/01/2019 | 2062900 |
|  | 01/04/2019 | 2054474 |
|  | 01/07/2019 | 2042930 |

**Note:** For each quarter, the population (n) reflects numbers for the overall dynamic cohort, and does not correspond to the number of individuals present in the cohort within any given year.
